# Prefrontal high definition cathodal tDCS modulates executive functions only when coupled with moderate aerobic exercise in healthy persons

**DOI:** 10.1038/s41598-021-87914-4

**Published:** 2021-04-19

**Authors:** Fabian Thomas, Fabian Steinberg, Nils Henrik Pixa, Alisa Berger, Ming-Yang Cheng, Michael Doppelmayr

**Affiliations:** 1 Department for Sports Psychology, Institute for Sport Science, Johannes Gutenberg-University Mainz, Albert Schweitzer Straße 22, 55128 Mainz, Germany; 2 School of Kinesiology, Louisiana State University, 1246 Pleasant Hall, Baton Rouge, LA 70803 USA; 3 Department for Neuromotor Behavior and Exercise, Institute of Sport and Exercise Sciences, Westfälische Wilhelms University Münster, Horstmarer Landweg 62b, 48149 Münster, Germany; 4 School of Psychology, Shanghai University of Sport, No. 650 Qingyuan Ring Road, Yangpu District, Shanghai, 200438 China

**Keywords:** Cognitive neuroscience, Human behaviour, Neurology

## Abstract

Transcranial direct current stimulation (tDCS) is a promising tool to enhance cognitive performance. However, its effectiveness has not yet been unequivocally shown. Thus, here we tested whether coupling tDCS with a bout of aerobic exercise (AE) is more effective in modulating cognitive functions than tDCS or AE alone. One hundred twenty-two healthy participants were assigned to five randomized controlled crossover experiments. Two multimodal target experiments (EXP-4: anodal vs. sham tDCS during AE; EXP-5: cathodal vs. sham tDCS during AE) investigated whether anodal (a-tDCS) or cathodal tDCS (c-tDCS) applied during AE over the left dorsolateral prefrontal cortex (left DLPFC) affects executive functioning (inhibition ability). In three unimodal control experiments, the participants were either stimulated (EXP-1: anodal vs. sham tDCS, EXP-2: cathodal vs. sham tDCS) or did AE (EXP-3: AE vs. active control). Participants performed an Eriksen flanker task during ergometer cycling at moderate intensity (in EXP. 3-5). Only c-tDCS during AE had a significant adverse effect on the inhibition task, with decreased accuracy. This outcome provides preliminary evidence that c-tDCS during AE over the left DLPFC might effectively modulate inhibition performance compared to c-tDCS alone. However, more systematic research is needed in the future.

## Introduction

Research on transcranial direct current stimulation (tDCS) suggests it has positive effects on neuroplastic processes and brain functions. Accordingly, it could potentially treat diseases associated with maladaptive neuroplasticity^1–3^ and provide value for other applied settings, such as modulating motor functions^4–6^, sports performance enhancement e.g.,^7^, sport neuro-diagnostics^8^, and in neuro-ergonomics/human factors contexts^9,10^. Despite these promising avenues, mechanistic pathways are well-studied but not yet completely understood^11^, and critics have emerged regarding the reliability of behavioral and physiological effects due to factors such as interindividual variability in response to tDCS^12,13^.

Previous studies have shown a single application of conventional tDCS (e.g., using two sponge electrodes, typically with a size of 35 cm^2^) over the dorsolateral prefrontal cortex (DLPFC)^14^, but also over multiple sessions^15^ can modulate cognitive abilities such as core executive functions (EF)^14^. However, within the cognitive domain, some meta-analyses have reported no effectiveness for a single session in healthy persons^12^, others have reported significant but weak effect sizes^16,17^. Thus, despite the problem of reliability, it is also necessary to enhance tDCS effects, which in turn might increase the likelihood of establishing meaningful clinical outcomes for its therapeutic or non-therapeutic usage. Two approaches that might improve the tDCS effects have been investigated frequently: increasing the current intensity and/or prolonging the stimulation duration. However, while increasing stimulation intensity and/or duration within certain limits enhances the effects of tDCS^18^, a modulation beyond these limits led to less predictable non-linear results regarding efficacy and directionality of tDCS effects^19,20^. Therefore, it is argued that the effects of conventional tDCS might be improved by technical or methodological advancements such as high-definition (HD) tDCS, multi-site stimulation^21^, or stimulation with different polarities in a specific sequence to initiate effects related to metaplasticity^22^. Indeed, some studies showed comparable modulating effects of HD-tDCS on executive functions^23–25^ and enhanced effects using a stimulation protocol where two sessions of the same or opposite polarity are applied with a short time interval in-between (a so-called metaplastic protocol)^26^. Others have proposed multimodal approaches, such as combining brain stimulation with physical exercise such as aerobic exercise (AE)^27–29^, specifically within the cognitive domain^29^. Indeed, there is emerging evidence that combined tDCS and AE (tDCS-AE) has greater effectiveness with reduced pain perception in fibromyalgia^30^. Combined tDCS-AE has also been shown to be associated with decreased inflammatory processes^31^ and reduced chronic pain^32^. Moreover, enhanced cognitive performance has been observed when tDCS is accompanied by a physical exercise program, including AE^15^. The rationale for combining tDCS and AE to modulate cognitive functions comes from recent empirical observations indicating remarkable similarities in their effects on brain activity (e.g., oscillatory activity), including the cognitive domain^28,29^.

For example, conventional a-tDCS over the prefrontal cortex (PFC) and especially over the DLPFC lasting 10–30 min improved inhibition (i.e., reaction times) in Flanker Tasks , Go/No-Go Tasks, Stop-Signal Tasks and Stroop Tasks paradigms^24,33–36^. Proposed tDCS effects on brain activity from a neurophysiological perspective include the modulation of resting-state activity, brain oscillations, brain perfusion, and oxygenation, bioenergetics, functional connectivity, event-related spectral perturbations (ERSPs), and event-related potentials^37^.

Comparable effects on brain activity with that of tDCS are well-known in the AE field. Compelling evidence exists that single bouts of aerobic exercise (AE) can temporarily alter neural excitability and cognitive functions both during or immediately following AE^38–41^. Acute AE increases the release of diverse neurochemical substances such as noradrenaline, adrenalin, dopamine, brain-derived neurotrophic factors (BDNF), and lactate^42^. The release of these substances, leads either to the optimal or suboptimal preparation of a person for action and aids in neurogenesis and neuroplasticity^42–44^. AE effects are moderated by several personal characteristics such as individual fitness level, age, sex, and methodological factors such as exercise intensity, duration, test timing, and outcome measure^43,45^. However, AE-induced alterations of cognitive functions seem to be reliable and most effective both during (between 20 and 60 min) and following AE (up to 60 min) with moderate AE intensities (e.g., in the range of 55–75% of maximal individual heart rate, or 40 to 60% of maximal oxygen uptake) in young, healthy persons^38,39,46^.

There are remarkable similarities between tDCS- and AE-induced effects on cognitive and brain functions and their ability to modulate neural excitability by a one-time application (e.g., only 20 min of either tDCS stimulation or AE). Therefore, in a recent review^29^ outlining both approaches' mechanistic pathways, we proposed that it might be beneficial to apply tDCS-AE within one session to enhance the effectiveness and synergize the effects of both methods. Nevertheless, neurophysiological or neurochemicals responses of combined approaches are not studied yet^29^. Thus, it remains somewhat speculative how the two approaches might specifically interact when coupled. As outlined in our recent review on this topic^29^, the modulation of EFs by anodal tDCS during stimulation (i.e., online) including HD-tDCS is attributable to changes in resting membrane potential through increasing cortical excitability^18,47,48^ and neurochemicals like dopamine involved in cognitive processes since EF tasks require the activation of the noradrenergic and dopaminergic pathways^49–51^. The catecholamine hypothesis postulates that the enhancement of EFs during AE is due to a release of catecholamine affecting several brain areas and functions. A simultaneous application of tDCS might specifically modulate the activity of the DLPFC, while AE may activate broader networks through reticular arousal activations pathways and neural oscillatory modifications through the release of catecholamine supporting executive function processing in a widespread cortical network including the DLPFC^29,52,53^. The involvement of the two pathways (exogenous tDCS and endogenous AE modulation) may then, as an example, enhance the EF inhibition in case of anodal tDCS (both upregulating activity) or reduce the inhibitory function in the case of cathodal tDCS (upregulating through AE and inhibiting through cathodal tDCS) respectively.

If the coupling of tDCS and AE works as proposed, combining tDCS and AE could be a promising avenue for several therapeutic (e.g., depression, stroke, or pain) and non-therapeutically approaches (e.g., sports or human factors). Since this proposal, three studies have tested whether tDCS-AE modulates cognitive functions. Hendy et al. (2019) investigated whether high-intensity AE on a cycle ergometer immediately before conventional tDCS application primes the brain to improve anodal tDCS-induced effects (anode over left DLPFC) on inhibition (Stroop test) and updating/working memory (n-back test)^54^. Hussey et al. (2020) stimulated the left DLPFC uni-hemispheric with conventional tDCS after 20 min of moderate AE running on a treadmill and tested inhibition (Flanker test), updating/working memory (n-back test), and sustained attention during brain stimulation^55^. Thomas et al. (2020) stimulated the left DLPFC with HD-tDCS during 20 min of moderate AE on a treadmill. They tested inhibition (Eriksen flanker test) and updating/working memory (2-back test) immediately after the HD-tDCS-AE sessions^56^. None of the three studies found any significant additional modulatory effect of cognitive functions when the combined approaches were compared to sham, inactivity, or single active applications. Common features across all three studies were DLPFC stimulation, testing core cognitive performance (i.e., the two EF inhibition and updating), and testing after AE.

Thus, no evidence exists that combining tDCS and AE might have additional acute benefits beyond those that are known for isolated applications. However, this only accounts for testing cognitive functions after an AE session with different intensities, and only our approach used HD-tDCS^56^ to increase focal stimulation^57,58^. Thus, one crucial factor that remains to be explored is testing cognitive functions during HD-tDCS-AE. Although other factors have differed between studies (e.g., tDCS montage or AE intensity), these need to be addressed in further systematic investigations. Therefore, in this study, we employed a "double-online" approach, i.e., tDCS application during exercising and cognitive task execution. We asked participants to perform an EF test (inhibition tested by a Flanker test) while receiving either HD anodal or cathodal tDCS over the left DLPFC and exercising on a cycle ergometer with moderate AE intensity. In two experiments, we tested whether combined tDCS-AE interventions modulate inhibitory control performance. In three additional experiments, we controlled for the single effects of a-tDCS, c-tDCS, and AE. Based on the knowledge so far that in most studies, a-tDCS improved and c-tDCS either decreased or had no effects on EF, we explored whether a-tDCS and AE would positively, and c-tDCS negatively affect EF. Additionally, we explored whether combined a-tDCS-AE or c-tDCS-AE has additive (a-tDCS-AE) or wash-out (c-tDCS-AE) effects on inhibition (i.e., no detrimental effects of c-tDCS on the executive function inhibition). In Thomas et al. (2020), we found some preliminary indications that our applied stimulation protocol (the same as we applied here) reduced the subjective experience of exertion during c-tDCS-AE. In accordance with all the indications of tDCS effects on subjective and objective markers of exercise performance^7,59–61^, we therefore also monitored the rating of perceived exertion (RPE), heart rate (HR), and power output as a marker of cycling performance.

## Results

**Figure 1:**
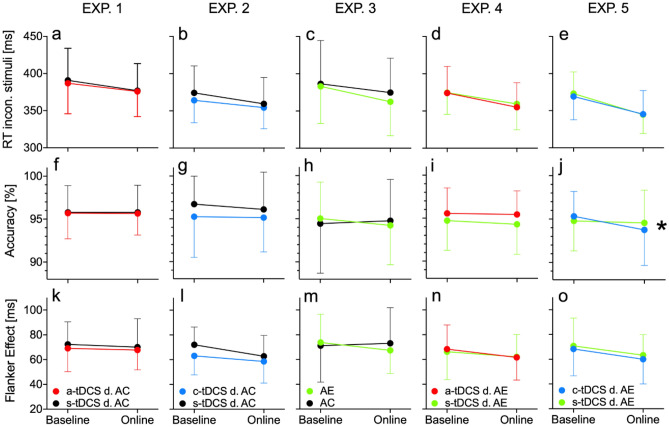
Results for the flanker task across experiments. Time × Condition interaction plots are displayed for the three variables reflecting Flanker test performance. The mean baseline and online values are plotted as the arithmetic mean and standard deviations. Statistical data are reported in the Result section. *asterisk displays a sig. (*p* < 0.05) interaction effect. AE = active control, AE = aerobic exercise. Figure drawn with GraphPad Prism version 8.2.1.

There were no baseline differences between conditions in cognitive performance in any of the variables and across experiments (all *p* > 0.05, for descriptive values, see Table A.3 in the supplementary material). Tables 1 and 2 show the ANOVA interaction statistics for EXP. 1 to EXP. 5 for the cognitive test data, and the physiological (HR and Watt) and subjective data (RPE). For Flanker task-related variables, a relevant interaction within EXP. 5, in which c-tDCS-AE was compared with s-tDCS-AE, indicated that c-tDCS-AE caused a significantly decreased response accuracy from pre- to post-testing compared to s-tDCS-AE with a large effect size (see Fig. 1j) (*F*(1,23) = 4.58, *p* = 0.043, $\eta_{p}^{2}$ = 0.17, post-hoc power 1 − β = 0.98). Bonferroni corrected post-hoc tests yielded only a significant (*p* = 0.045) performance decrease between Baseline c-tDCS-AE compared to online c-tDCS-AE, but no other significant pairwise comparison (all *p* > 0.05). For all other possible Time × Condition effects, no significant interaction emerged (all *p* > 0.05, see Table 1). However, one statistical trend (*p* = 0.066) with a high effect size emerged for the flanker effects in EXP. 3, indicating a slight performance increase during AE compared to that of AC (*F*(1,22) = 3.74, *p* = 0.066, $\eta_{p}^{2}$ = 0.15, see Fig. 1c).

**Table 1:** Statistical key figures of the flanker task-related dependent variables. Only the values for Time × Condition interactions are shown. AC = active control, AE = aerobic exercise, RA = Response accuracy, RT = Reaction time

Experiment	N	Dependent Variable	df	F	*p*	$\eta_{p}^{2}$
EXP. 1: a-tDCS vs. s-tDCS during AC	21	RT incon. stimuli	1.20	0.5	0.49	0.02
		RA	1.20	0.0	0.95	0.0
		Flanker effect	1.20	0.04	0.84	0.01
EXP. 2: c-tDCS vs. s-tDCS during AC	17	RT incon. Stimuli	1.16	0.83	0.36	0.05
		RA	1.16	0.15	0.70	0.01
		Flanker effect	1.16	1.1	0.32	0.06
EXP. 3: AE vs. AC	23	RT incon. stimuli	1.22	2.75	0.11	0.11
		RA	1.22	0.84	0.36	0.04
		Flanker effect	1.22	3.74	0.06	0.15
EXP. 4: a-tDCS vs. s-tDCS during AE	16	RT incon. stimuli	1.15	0.56	0.47	0.04
		RA	1.15	0.15	0.70	0.01
		Flanker effect	1.15	0.22	0.65	0.01
EXP. 5: c-tDCS vs. s-tDCS during AE	24	RT incon. stimuli	1.23	1.84	0.19	0.07
		RA	1.23	4.58	0.04	0.17
		Flanker effect	1.23	0.05	0.82	0.00

**Table 2:** Statistical values of the exercise-related dependent variables. Only the values for the Time   × Condition interactions are shown.

Experiment	*N*	Dependent variable	df	*F*	*p*	$\eta_{p}^{2}$
EXP. 4: a-tDCS- vs. s-tDCS during AE	16	RPE	2.50	0.82	0.47	0.05
		HR	2.28	0.56	0.60	0.03
		Watt	2.14	2.78	0.07	0.14
EXP. 5: c-tDCS vs. s-tDCS during AE	17	RPE	2.61	0.97	0.40	0.05
		HR	1.97	0.56	0.69	0.03
		Watt	1.76	0.67	0.50	0.03

**Figure 2:**
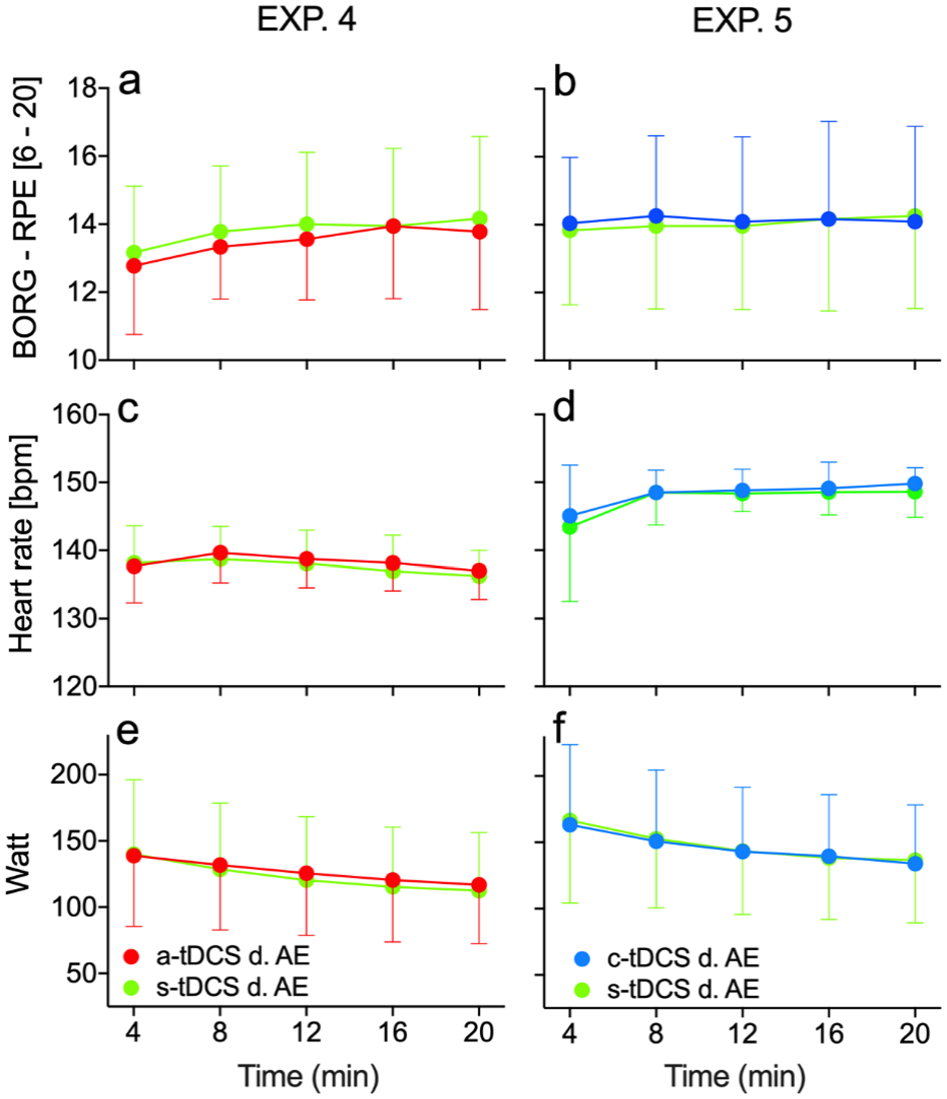
Exercise-related parameters RPE, HR, and Watt for EXP. 4 and EXP. 5 during the time course of the experiment. Presented are the mean values (error bar = SD) of the measuring points for BORG-RPE, heart rate, and Watt (pedal resistance) for EXP 4 (**a**, **c**, **e**) and 5 (**b**, **d**, **f**). Figure drawn with GraphPad Prism version 8.2.1.

Concerning the impact of combined tDCS-AE experiments on RPE, HR, and Watt, we could not find any relevant interaction in EXP. 4 or EXP. 5 (see Fig. 2 and Table 2). However, a large effect size with a statistical trend emerged for a-tDCS-AE when compared with s-tDCS-AE (EXP. 4). In EXP. 4, under the influence of a-tDCS, the participants seemed to be slightly more capable of cycling with elevated pedal resistance (W) than they did under the influence of s-tDCS (*F*(2.14,36.39) = 2.78, *p* = 0.072, $\eta_{p}^{2}$ = 0.14, see Fig. 2e). All main and interaction ANOVA effects for the entire data set are shown in the supplemantary material (Tables A.3 to A.7).

**Table 3:** Descriptive anthropometric data in all five experiments. AC = active control, AE = aerobic exercise. Mean ± standard deviation

Experiments	N (male, female)	Age	Height (cm)	Weight (kg)
a-tDCS vs. s-tDCS during AC (EXP. 1)	21 (10, 11)	25.7 ± 2.2	173.9 ± 11.7	72.5 ± 15.5
c-tDCS vs. s-tDCS during AC (EXP. 2)	24 (12, 12)	25.2 ± 1.7	176.9 ± 9.7	70.0 ± 9.6
AE vs. AC (EXP. 3)	24 (15, 9)	25 ± 2.5	175.5 ± 9.4	69.6 ± 11.0
a-tDCS vs. s-tDCS during AE (EXP. 4)	22 (11, 11)	25.5 ± 2.1	172.9 ± 9.6	70.2 ± 12.4
c-tDCS vs. s-tDCS during AE (EXP. 5)	24 (15, 9)	24.9 ± 1.5	177.6 ± 9.2	74.3 ± 11.4

## Discussion

This study investigated whether HD-tDCS, applied concurrently with moderately intense AE in healthy young people, has any beneficial effect on inhibition performance beyond the effects that have been previously reported when each (either tDCS or AE) is applied alone. Five experiments were conducted that contrasted a-tDCS-AE or c-tDCS (left DLPFC) with s-tDCS-AE sessions while controlling for single applications (i.e., only AE, a-tDCS, or c-tDCS). Three critical results emerged: First, neither a-tDCS nor c-tDCS nor AE applied in isolation had any clear significant effect on flanker test performance when tested during AE, except for a trend (*p* = 0.066; $\eta_{{\text{p}}}^{{2}}$ = 0.15) for AE with a slightly decreased flanker effect. Second, when a-tDCS was coupled with AE, no effects were elicited. Third, when c-tDCS was coupled with AE, a significant decrease in the accuracy of the flanker test indicated a modification of inhibition performance. In contrast, neither AE nor c-tDCS alone had any such effect on accuracy. This pattern suggests that c-tDCS over the left DLPFC only modulates cortical activity when administered during AE, possibly due to the specific brain state associated with AE.

The null effects of applying tDCS alone confirm our recent study in which the same protocol was used offline (i.e., cognitive testing immediately after tDCS-AE)^56^. However, it remains controversial to the widely accepted assumption that one single treatment of tDCS has significant effects on cognitive performance^14^. The majority of tDCS studies have shown that a-tDCS and c-tDCS elicit excitatory effects with increased performance and inhibitory effects with decreased performance, respectively^35^. Although the dichotomy between anodal and cathodal polarity is well established in the motor domain, and not necessarily for the cognitive domain^35^, there is some evidence that this also accounts for the stimulation of prefrontal brain structures and, thus, cognitive functions^37^.

In a neuroimaging review by Wörsching et al. (2016), it has been outlined that several neurophysiological measures such as the resting-state connectivity, event-related potentials, and functional magnetic resonance imaging (fMRI)-based activation patterns are affected by prefrontal tDCS. Thereby, they review three independent prefrontal tDCS studies^62–64^ on cognition, including executive functions, electrophysiological measures, and neuroimaging that show polarity-dependent effects indicating an anodal excitatory, and a cathodal inhibitory effect on cortical activity^37^. New research by Dennison et al. (2019) showed that cathodal stimulation of the DLPFC impaired cognitive flexibility (reversal learning, but not task switching, an executive function)^65^. Importantly, cathodal tDCS administered in combination with tyrosine, a precursor of dopamine, reversed the adverse effects. Additionally, there is evidence that one session of bifrontal tDCS over DLPFC increases extracellular dopamine levels in the striatum^66^, and it is well-known that dopamine is involved in executive functions through the meso-cortico-limbic pathway^66,67^ This indicates a decisive role of the dopaminergic system in tDCS-induced effects on cognitive systems. Therefore, we expected improved cognitive test performance during a-tDCS due to increases in excitability and, most likely detrimental performance during c-tDCS due to decreased excitability. However, we, and also some other studies, observed no effects of a single application of a-tDCS or c-tDCS ^cf.^^68^. In our recent study, we discussed the lack of an effect of a-tDCS on inhibition ability by attributing the lack of any effects to different stimulation protocols^56^, in contrast to studies identifying improved inhibition due to a-tDCS of the left DLPFC^33,34^. Those used a different and conventional tDCS montage, which might have provoked stimulation of other brain areas such as the anterior cingulate cortex (ACC) in addition to the left DLPFC. The ACC is a critical brain area involved in inhibition processing^69^, and thus, its modification might have provoked the enhanced inhibition performance rather than modification of the left DLPFC. Consequently, the present and our former study suggest that a focal HD-a-tDCS of the left DLPFC is insufficient to modify inhibition, at least with parameters used here.

There are likely many other interdependent factors contributing to this lack of replicability. Publication bias^35^, inconsistent stimulation protocols and inter-individual variables such as neuroanatomy, sex, age, neurochemistry, and health status, which all come with state-based dependent factors such as vigilance, activation before (i.e., priming), and task-dependent factors, may contribute^70–72^. Furthermore, as outlined above on a neurophysiological level, several studies have shown controversial tDCS polarity and task-specific effects, even with improved cognitive performance with c-tDCS^14,73–75^.

One might also argue that a different tDCS protocol (e.g., higher current, longer stimulation duration, different montage, use of conventional tDCS) would have produced more conclusive results. However, the study situation on this is inconsistent. Dubreuil-Vall et al. (2019) and Angius et al. (2019) showed improved inhibition with 2 mA current, 30 min stimulation duration, and the same montage (stimulation electrode = F3, return electrode = Fp2)^33,34^. But, Dubreuil-Vall et al. (2019) used HD-tDCS (electrode size = 3.14 cm^2^) and Angius et al. (2019) conventional tDCS (electrode size = 35 cm^2^). These results suggest that our used current intensity might have been too weak and the stimulation duration was too short. However, in a study by Hoy et al. (2013) using conventional tDCS with the same montage as the aforementioned studies, only 1 mA of current with a stimulation duration of 20 min resulted in a better 2-back performance (a cognitive test to measure working memory), while 2 mA had no effect. From this, we conclude that current intensity is not necessarily the major factor contributing to the appearance of modulations (also see Esmaeilpour et al., 2018). In addition, and unlike all the studies mentioned, we did not use the conventional 2-electrode montage but a 4 × 1 ring configuration which might stimulate the left DLPFC more focal. As discussed above, other brain areas which are also crucial for inhibition might have been modulated. For example, Hogeveen et al. (2016) compared the effect of conventional and HD-tDCS montages on response inhibition targeting the inferior frontal cortex (IFC). Both HD- and conventional tDCS improved response inhibition. With the placement of the return electrode on the contralateral supraorbital region Dubreuil-Vall et al. (2019) and Angius et al. (2019) might also have stimulated other crucial brain areas (e.g., the IFC or the ACC see Nee et al.^69^), which might be a possible explanation why we could not replicate those findings.

As in our previous study, we could not unequivocally replicate the well-known AE effects on cognitive functions^76^, which we attributed to factors such as exercise modality, AE intensity, and rather poorly defined individual exercise specifications^56^. However, in the current approach, we used almost the same exercise intensity (i.e., moderate AE with a slight increase in the HR range by 5%). Still, we changed the exercise modality (cycling instead of running) and testing time (during AE versus after AE). However, here we observed a significant trend (i.e., *p* = 0.066) indicating that AE improved inhibition, including a high effect size ($\eta_{p}^{2}$ = 0.15), which would confirm the well-known positive effects of moderate AE intensity on cognition reported in the literature^38,39^. More specifically, as outlined in the introduction, it is well-known that one session of acute exercise can modulate cognitive and motor functions. The effects range from core cognitive functions such as inhibition or task switching as EF, working memory to higher-order cognitive functions such as complex planning and motor learning processes^29,38,42^. For example, in a study by Davranche et al. (2009), the reaction times during moderate aerobic exercise (cycling at 50% of maximal aerobic power) decreased significantly compared to inactivity^77^. We could not replicate these results in EXP. 3 although a statistical trend in the interaction effect of the flanker effect might be a possible indication for improved response inhibition (see Fig. 1, cell m). This could be due to the used intensity calculation based on the formula “HR_max_ = 220–age”. In the present study, the majority of the participants rated the intensity as "moderate “ (59%) , but there were also participants who rated the intensity as "high" (27%) or even "low" (7%) at the same percentage HR_max._ Davranche et al. (2009) determined the physical capacity of their subjects in advance using spiroergometry. This allowed them to define and control the intensity more individually. Thus, future research in the context of coupling tDCS-AE should improve intensity definition to reduce interindividual differences to better account for the intensity-dependent effects of AE-induced effects on executive function.

If AE effects are stable, a trend or a significant finding should also have emerged when AE was coupled with a-tDCS (EXP. 4), which was not the case (no effect; see Fig. 1d, i, n). If true, it would suggest that adding a-tDCS to AE has an adverse impact on cognitive performance during AE since any positive effects elicited by AE (i.e., the trend in EXP. 3) were disrupted by adding a-tDCS to AE in EXP. 4. In this context, other studies observed that in some cases, a-tDCS over the prefrontal cortex (PFC) reversed the effects on Stop Signal Tasks measuring impulsivity, but also the executive function inhibition in participants with high trait impulsivity that correlates with dopamine levels. This suggests interindividual differences in personality and neurochemistry^78^. Additionally, metaplasticity tDCS protocols changed the polarity-dependent effects of c-tDCS, i.e., a 10-min preconditioning a-tDCS phase increased working memory performance during a subsequent 10-min c-tDCS condition after a 10 min rest. At the same time, this was not the case for a-tDCS^26^. Only a slight non-significant (*p* < 0.10) elevation of inhibition by AE in our Exp-3 and the non-significant combined a-tDCS-AE effects do not provide compelling evidence that AE with a-tDCS interacted comparably (e.g., dopamine overshoot or metaplasticity). Hence, a-tDCS might not have reversed or interfered with any AE-induced positive effects on inhibition. Thus, our data indicate that the used a-tDCS-AE protocol does not initiate any additive or synergetic effects because it does not elicit any clear (except the statistical trend for EXP. 3) effects when applied alone (EXP. 1 and EXP. 3). From this, we can conclude that under the circumstances administered here (moderate AE intensity, 1 mA, left DLPFC HD-tDCS for 20 min, flanker test), a-tDCS-AE does not modulate inhibition performance.

We found that although c-tDCS applied alone (EXP. 2) did not modify cognitive performance, the c-tDCS-AE administered in EXP. 5 provoked a significant decrease in the accuracy performance of the Flanker test. This discrepancy between our former study (offline c-tDCS and c-tDCS-AE application yielded no effects) and the no-effects of EXP. 2 could be attributed to the high variability of the tDCS effects^79^. However, this requires further research, for example, using individual head models to optimize the stimulation parameters. For example, a study by Filmer et al. (2019) showed that the efficacy of tDCS to prefrontal areas is related to underlying cortical morphology^80^. Cortical thickness of the left (but not right) PFC accounted for almost 35% of the variance in stimulation efficacy across participants providing evidence that cortical morphology is related to an individual's behavioral response to tDCS. This implies that a generalized tDCS protocol has a different efficacy for each participant due to their different brain anatomical characteristics’^81,82^. Future studies should use structural magnetic resonance imaging (MRI) scans to create individual 3D head models for these calculations, effectively predicting current densities in individual brains^83^. Consequently, individualized MRI-based stimulation dosage adjustment (e.g. current intensity and/or electrode placement) might be considered instead of one-size-fits-all approach.

However, if the c-tDCS effect remains consistent across other stimulation protocols, it could have a decisive impact on future research. Given our behavioral approach and the nonexistence of neurophysiological parameters when combining tDCS-AE, we can only speculate about the mechanistic interactions and why c-tDCS might be effective when coupled with AE. Numerous studies have reported AE-induced neural activity changes such as neural excitability, cerebral blood flow, oscillatory activity, resting state, and event-related potentials^29,42^. It is also known that AE can induce the synthesis and release of diverse neurochemical substances, including brain-derived neurotrophic factors, noradrenaline, adrenaline, serotonin, dopamine, norepinephrine, epinephrine, glutamate, and GABA^29,42,84^. Therefore, there is no doubt that during and following AE, the brain is in a unique state, and thus, might be more susceptible to exogenous modification acting on comparable mechanistic pathways^27–29^. Accordingly, the additional modulation of the left DLPFC by c-tDCS in EXP. 5 might have made it more likely that a slight downregulation occurs. Downregulation would include performance decreases in tasks involving inhibitory control, where it is well-known that left DLPFC structures are involved^52,85,86^. It remains to be determined whether this pattern is constant and why AE can modify only c-tDCS but not a-tDCS effects. However, if replicable and occurring in other situations, coupled c-tDCS-AE might be helpful, for instance, for treating pain disorders where the DLPFC is involved in the cognitive aspect of pain^87^, migraine^88^, stroke^2,89,90^ or focal epilepsy^91^.Unlike some previous studies and our recent study, neither a-tDCS nor c-tDCS during AE affected perceived exertion. This is not surprising since the modulation of perceived exertion through tDCS in physical exercise settings is not consistent in the literature^61^, possibly due to the discussed moderating parameters. In the present study, we modified the AE modality from our previous study^56^; due to online cognitive testing, cycling was more feasible than running. The same c-tDCS protocol slightly decreased perceived exertion during moderate-intensity running^56^, but not during cycling, suggesting that the effects of tDCS on perceived exertion are dependent on the exercise modality, which should be evaluated in further studies. Alternatively, this unstable outcome additionally underlines the low inter- and intrasubject reliability of tDCS. Thus, it may be that the correct protocol must always be configured for each person and application individually. Biomarkers, individual head models and electrical field modeling, or cluster analysis might help to predict/optimize the effectiveness of tDCS in the future and when combined with AE protocols^34,92^. Given this is one of the first conducted studies in this complex context, several limitations require further consideration. First, we did not employ a complete within-subject design to test the various conditions. Instead, we had five within-subject experiments with five different participant groups. This might have provoked that interindividual variability of tDCS and AE, respectively, blurred any possible single treatment as well as coupled tDCS-AE effects and limited direct comparisons between conditions of different experiments. However, due to the high amount of conditions we tested, a complete within-subject design would have been challenging and would come with other limitations. Second, we used a simple formula that was not adjusted to individual fitness levels to calculate the HR range and define the AE intensity levels individually. This might have resulted in varying intensities across participants and thus influenced cognitive performance. Third, the effects of the 4 × 1 tDCS montage are not yet established in the literature. However, we chose this setting to ensure increased focal targeting of the left DLPFC. Fourth, we found only a significant decrease in accuracy in the range of a few percent, such as it remains to be determined whether this has a real-life consequence. Fifths, we had only 48 h in-between tDCS session, a time phase which could be longer in future studies (e.g., above one week).

More research that considers neurophysiological and brain plasticity markers, inter-individual differences, including full blinding of polarity (see methods that experimenter was blinded of whether real or sham tDCS, but not whether anodal or cathodal polarity) is warranted to shed more light on the effects of coupling tDCS with AE. Additionally, several parameters that were not tested here should be considered in future research. The use of different tDCS and AE intensities, multiple sessions of tDCS and AE, duration of AE and tDCS, with respect to online and offline effects, other cognitive tasks (e.g., working memory), motor tasks, and involving meta-plastic study designs (i.e., inserting time delays between tDCS intervention and AE) should be tested to explore whether tDCS-AE might be a reliable tool to increase tDCS-induced effects.

## Conclusion

Neither a-tDCS nor c-tDCS over the left DLPFC, nor AE alone, had any apparent significant effect on inhibition performance, which is in contrast to most studies that suggest a one-time application of tDCS or a bout of AE can modulate cognitive functions. However, coupling c-tDCS with AE resulted in decreased accuracy of an inhibitory control task that may be related to impaired left DLPFC activity. This provides some preliminary evidence that cathodal HD-tDCS on the left DLPFC with 1 mA might only modulate executive function (i.e., inhibition) when applied during moderate AE, possibly due to the unique brain state elicited by AE. Due to the exploratory character and lack of mechanistic evidence/explanations along with many moderating parameters, this assumption requires further systematic research and might only account for the specific parameters applied here. However, it opens an exciting avenue for further investigation of tDCS-AE approaches in other domains, participant groups, other cognitive functions, and with modified parameters.

## Material and methods

### Participants

One hundred twenty-two healthy adults were recruited to participate in the study (anthropometric data, Table 3). Data of seven participants were excluded due to technical issues during the experiments. The remaining one hundred fifteen participants (63 males, 52 females) were assigned to five experiments, and anthropometric data are detailed in Table 3. Considerations of sample size included comparing average sample sizes of recent studies on acute tDCS and AE effects on executive functions (78 studies with an average of n = 22 in^14,45^), and statistical power was additionally calculated post-hoc for significant interaction effects. All participants gave their informed consent before participating and in accordance to international guidelines for tDCS research^93^ were asked to disclose preexisting neurological and psychological conditions, medical conditions, drug intake (mainly central nervous system acting medication), alcohol/tobacco consumption per week and caffeine intake during the previous week. Contrary indicators for tDCS included^93^: diagnosed epilepsy, seizure, traumatic brain injury, cochlear implant, implanted neurostimulator, metal in the brain area, pregnancy or possibility of pregnancy, heart surgery, pacemaker or cable in the heart, head or brain surgery, infusion device for medication. Participants that responded with yes to one of the listed conditions were excluded from participation. The study was conducted in accordance with the Declaration of Helsinki, and the protocol was approved by the ethics committee of the Johannes Gutenberg-University, Mainz.

### Transcranial direct current stimulation (tDCS)

**Figure 3:**
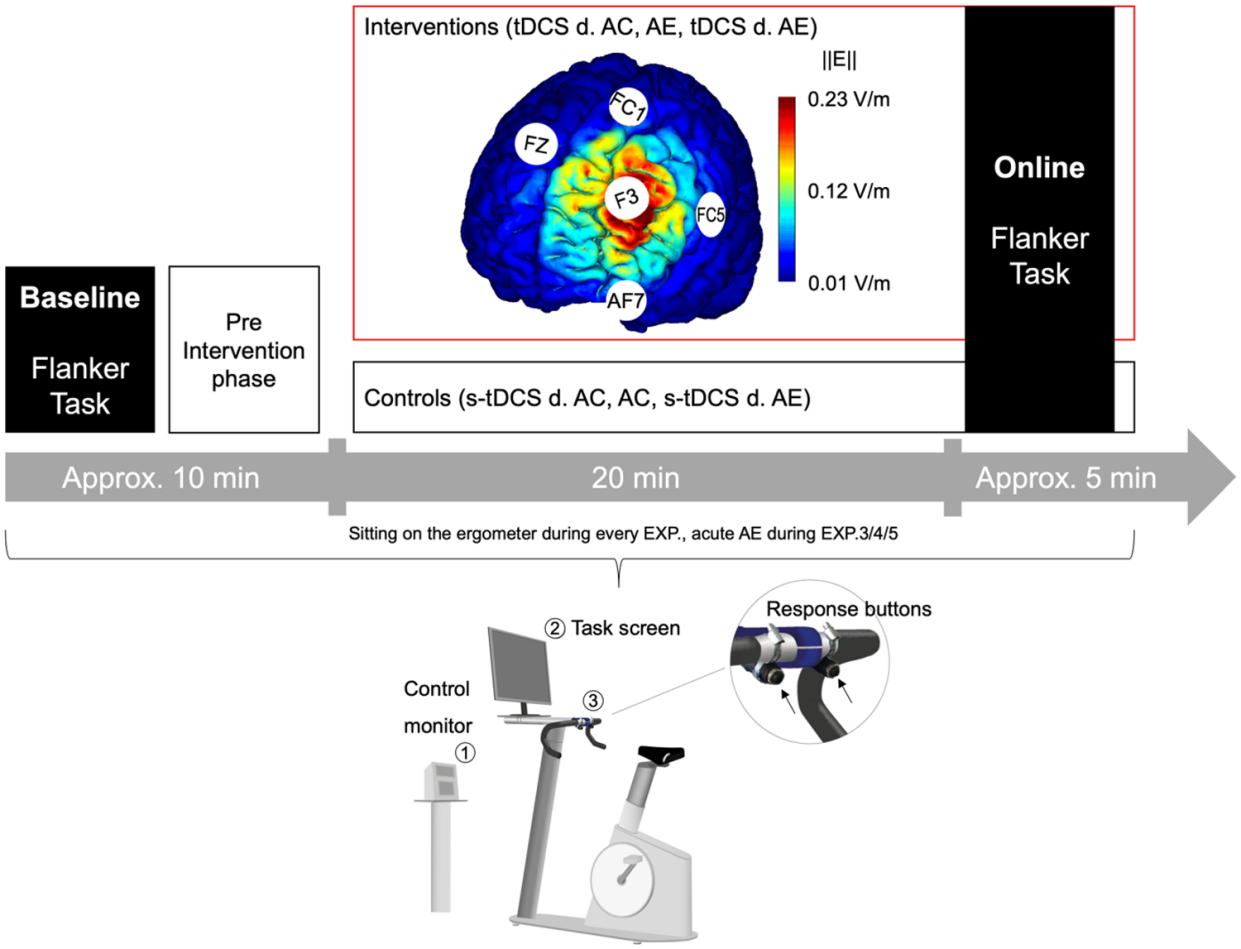
Experimental design and timeline, tDCS montage, and cycle ergometer with cognitive test set-up. The upper part shows the computational model of the electric field (||E||) acting on the left DLPFC of the participants during anodal and cathodal tDCS (Neuroelectrics Instrument Controller version 2.0.10, Neuroelectrics, Barcelona, Spain). The middle part shows the experimental timeline comprising the intervention (EXP. 1: a-tDCS alone, EXP. 2: c-tDCS alone, EXP. 3: AE alone, EXP. 4: a-tDCS during (d) AE and EXP. 5: c-tDCS during AE) and control conditions (EXP 1.: s-tDCS alone, EXP. 2: s-tDCS alone, EXP. 3: Active control (AC), EXP. 4: s-tDCS during AE and EXP. 5: s-tDCS during AE). Active control (AC) was applied in all control conditions, which means that even during s-tDCS, all participants sat on the cycle ergometer and cycled with a very low intensity of 15 Watts. The lower part shows the cycle ergometer with 1) the control monitor displaying to the investigator the heart rate, pedal cadence, and Watt; 2) the task screen where the cognitive tests were presented during cycling; and 3) the response buttons (The Black Box Toolkit) fixed at the steering of the ergometer, enabling participants to easily make responses by button presses without changing posture. Cycle ergometer drawing was done using the software SketchUp.

tDCS was applied using a wireless, computer-controlled device (StarStim, Neuroelectrics, Barcelona, Spain). The montage comprised five high-definition (HD) stimulation electrodes (Ag/AgCl, 3.14 cm^2^) in a 4 × 1 ring configuration targeting the left DLPFC (^56^ see Fig. 3).

Current was applied with an intensity of 1 mA (-1 mA for c-tDCS) via the stimulation electrode over 20 min before the cognitive test. During the cognitive test, the stimulation was maintained for the test’s duration (approximately 5 min; see Fig. 3). s-tDCS comprised 30 s of active stimulation at the beginning and at the end of the intervention (for all additional parameters, see Table 4).

**Table 4:** tDCS parameters for four of the five experiments. CI = current intensity; CD = current density.

Parameters	a-tDCS	c-tDCS	s-tDCS
Duration	~ 25 min	~ 25 min	~ 25 min
Ram-up/down	30 s	30 s	30 s
CI stim. electrode (CD)	1 mA (0.318 mA/cm^2^)	− 1 mA (− 0.318 mA/cm^2^)	–
CI return electrodes (CD)	− 0.250 m (− 0.08 mA/cm^2^)	0.250 mA (0.08 mA/cm^2^)	–
Electric field ||E||	0.23 V/m	0.23 V/m	–
Impedance cut-off	< 10 kΩ	< 10 kΩ	< 10 kΩ
Electrode size	3.14 cm^2^	3.14 cm^2^	3.14 cm^2^
Electrode material	Ag/AgCl	Ag/AgCl	Ag/AgCl
Dosage of applied current	1530 mC	1530 mC	35 mC
Applied in	EXP. 1, 4	EXP. 2, 5	EXP. 1, 2, 4, 5

The rationale of this protocol is based on three reasons. First, an increase in current intensity does not necessarily boost tDCS outcomes, as already described in the introduction. Therefore, we decided not to use the highest current intensity available for our device. 1 mA, -1 mA respectively, of current intensity, modulated EFs even in studies using conventional tDCS^63,94,95^, and in HD-tDCS studies^24,96^. The enhanced focal stimulation of the used 4 × 1 HD-tDCS ring configuration ensures high current densities, mainly in the target area. Compared to conventional tDCS, the risk of side effects is reduced as stimulation of non-target brain areas is kept to a minimum^97^. Second, we wanted to stimulate during AE and the flanker task. Therefore, the duration of the stimulation was chosen to match the duration of AE. In addition, stimulation durations of 10 to 30 min are typical stimulation durations that are used by many studies to modify EF^98^. Third, higher current densities might come with increased stimulation sensation, compromising the blinding procedure.

A double-blind setting ensured unbiased effects, i.e., both the investigator and the participants did not know whether active or sham tDCS is applied. However, since the five experiments were performed in a sequence, the investigator knew which experiment is currently running in terms of tDCS polarity (anodal or cathodal). Thus, the blinding accounts only for active versus sham, but not which polarity was applied in the case of active tDCS.

### Aerobic exercise condition

Participants cycled on a stationary ergometer (Lode, Netherlands) for approximately 25 min between 70 and 75% of maximum heart rate (HR_max_). HR_max_ was calculated using the formula "HR_max_ = 220—age"^99^. The intensity range was chosen based on the guidelines of the American College of Sports Medicine^100^ (moderate intensity = 65% and 75% of HR_max_). HR was measured using a chest belt (H3 heart rate sensor by Polar) and recorded by a training computer (RS800cx by Polar). During a five-min warm-up phase, the participants' HR was brought into the target range by continuously increasing the pedal resistance. After completing the warm-up, the investigator manually ensured that the HR was within these limits by increasing or decreasing pedal resistance via the control monitor (Fig. 3). The target cadence was 60 revolutions per min. If a participant cycled too fast or too slow, they were verbally corrected by the investigator. Pedal resistance, pedal cadence, HR, and time were displayed only to the investigator. The effects of AE were controlled by cycling with a pedal resistance of 15 W (W) with a comfortable cadence, which we called the "active control (AC)" condition. We assumed that this protocol has negligible to no exercise-specific effects on inhibition because of its very low intensity^101^. For better comparability between experiments, the AC condition was applied to all conditions that did not include AE (Fig. 1). During AE sessions (EXP. 4, and 5), the participants were asked to rate their perceived exertion on a scale ranging from 6 (no perceived exertion) to 20 (maximal perceived exertion) every 4 min^102^. The rating was realized with an on-screen visual fade-in of the scale for a few seconds during the documentary they watched on the task screen (Fig. 3), and the participants had to indicate a number corresponding to the perceived exertion to the investigator.

### Cognitive testing

We used the same flanker task as in our previous publication^56^. The flanker task is a typical task demanding response inhibition. Participants are required to focus on a target arrow and ignore surrounding arrows (i.e., the flankers) that could be congruent (i.e., >>>>> or <<<<<) or incongruent (i.e., <<><< or >><>>). Increased reaction times and decreased accuracy are usually observed for incongruent compared to congruent stimuli, representing the cognitive effort to inhibit the intuitive response^103^.

This task primarily comprises two blocks containing 52 stimuli each (52 incongruent and 52 congruent stimuli). The stimuli were randomized with equiprobable directionality with congruent and incongruent arrays of 3-cm-tall white arrows on a black background screen. Each array was presented for 600 ms with a randomized intertrial interval between 1000 and 1600 ms. The flanker task was presented on the task screen (Fig. 3) using the stimuli control software Presentation (Version 18.0, Neurobehavioral Systems).

### Experimental procedure

We designed five identical experiments (in terms of experimental tasks and timeline), and the protocol used a crossover design. Each of the five experiments was divided into two randomized, counterbalanced sessions (i.e., treatments) separated by at least 2 days and a maximum of 7 days. There are currently no standardized guidelines on the amount of time that should be left between tDCS sessions to ensure that any stimulatory effects have “washed out”. Although a study by Boggio et al. (2007) recommended having at least a week between testing sessions^89^, recent findings by Dedoncker et al. (2016) found no significant influence of the interval between sessions on cognitive outcomes for prefrontal tDCS^104^. Therefore, and in addition to test economy reasons and to account for AE carry-over effects, we decided on this range between sessions. The second measurement appointment on another day was only allowed to occur within a range of ± 2 h from the time of the first appointment to minimize the influence of circadian rhythm. Each session consisted of a baseline and an online measurement of the flanker task. Between measurements, the participants underwent the respective experimental intervention, as shown in Fig. 3. During every intervention, the participants watched a commercially available video documentary about Germany's landscape.

As indicated in Fig. 3 and Table 3, we conducted five experiments as in Thomas et al. (2020) that were all performed in the described way: In EXP. 1, we tested whether inhibition performance is positively affected by a-tDCS over the left DLPFC compared to sham tDCS (s-tDCS). In EXP. 2, we tested whether c-tDCS over the left DLPFC reduces inhibition performance compared to s-tDCS. In EXP. 3, we tested whether moderate AE intensity improves inhibition performance compared to inactivity. In EXP. 4, we explored whether combined a-tDCS-AE improves inhibition performance compared to s-tDCS-AE. In EXP. 5, we investigated whether the combined effects of c-tDCS-AE either maintained or reduced inhibition performance compared to s-tDCS-AE.

### Data analysis

We analyzed reaction time (RT) for incongruent stimuli and response accuracy (RA), defined as the mean percentage of correct responses in all trials in the flanker task. RTs > 1000 ms and < 100 ms were excluded from further analysis. Additionally, we calculated the so-called "flanker effect" for RT, which is the difference in RT between incongruent and congruent stimuli^105^. Every four min, the RPE was assessed verbally, and the current pedal resistance (in Watts) was noted. The training computer continuously recorded the HR at a sampling rate of 1 Hz. To determine the corresponding HR and cycling power in W, the means ± 30 s around the given Borg RPE/pedal resistance values were taken for further analysis and graphical presentation. Normal distribution of all variables where visually checked using Q-Q-plots and analyzed using the Shapiro–Wilk test (see appendix, table A2). In the case of normal distribution paired t-tests and in the case of non-normal distribution Wilcoxon tests were used to check for any baseline differences between conditions. As the analysis of variances is thought to be robust against violations of normal distributions^106,107^, two-way ANOVAs with repeated measures (rmANOVA) were performed for each dependent variable in every experiment using the R-based software jamovi^108^ (jamovi project, version 1.9). The ANOVA factor "Time" for the Flanker task consisted of baseline vs. online levels; for the RPE, HR, and Watt it consisted of 4 min steps recorded within 20 min of AE (4, 8, 12, 16, and 20 min). In all ANOVA analyses across EXP. 1 to EXP. 5, the factor "Condition" included the within-subject level treatment vs. control. Greenhouse–Geisser adjustments were applied when appropriate. Estimated effect sizes were reported using partial eta-square ($\eta_{p}^{2}$) values^109^. We excluded all outliers that differed by more than three standard deviations (SD)^56,110^ from the mean of each experiment (see Table 5). Because of the series of different parameters and experimental conditions, we will only report relevant significant Time × Condition interactions in the results section to ensure a good overview of the results. All ANOVA main and interaction effects are summarized in the supplementary material. For significant interactions, we also report post-hoc power using G*Power.

**Table 5:** Sample sizes before and after SD filtering and drop-outs for Flanker task and performance-related measures. AC = active control, AE = aerobic exercise.

Experiments	N (before SD filtering)	N flanker task (after SD filtering)	RPE/HR/Watt (after SD filtering)
a-tDCS vs. s-tDCS during AC (EXP. 1)	21	21	–
c-tDCS vs. s-tDCS during AC (EXP. 2)	24	17	–
AE vs. AC (EXP. 3)	24	23	–
a-tDCS vs. s-tDCS during AE (EXP. 4)	22	16	16
c-tDCS vs. s-tDCS during AE (EXP. 5)	24	24	17

## Supplementary Information

Supplementary Information

## Data Availability

The datasets generated during and/or analyzed during the current study are available from the corresponding author on reasonable request.
