# Supplementary material for: Prefrontal high definition cathodal tDCS modulates executive functions only when coupled with moderate aerobic exercise in healthy persons
Source: Sci Rep. 2021 Apr 19;11:8457. doi: 10.1038/s41598-021-87914-4 (PMC8055664; doi:10.1038/s41598-021-87914-4)
Supplement: Supplementary file 1 — Supplementary Information [file 41598_2021_87914_MOESM1_ESM.docx]

Supplementary Material

High definition (HD) cathodal tDCS, but not HD-anodal tDCS, targeting the left DLPFC, modulates executive function only when coupled with moderate aerobic exercise in healthy persons

Authors: Fabian Thomas^1#^ Fabian Steinberg^2*#^, Nils Henrik Pixa^3^, Alisa Berger^1^, Ming-Yang Cheng^4^, & Michael Doppelmayr^1^

^1^Institute for Sport Science, Department for Sports Psychology, Johannes Gutenberg-University Mainz, Albert Schweitzer Straße 22, 55128 Mainz, Germany, [fabianthomas27@icloud.com](mailto:fabianthomas27@icloud.com), aberge03@uni-mainz.de and [doppelma@uni-mainz.de](mailto:doppelma@uni-mainz.de)

^2^School of Kinesiology, Louisiana State University, 85 Huey P. Long Field House, Baton Rouge, LA 70803, USA, fsteinberg@lsu.edu

^3^Institute of Sport and Exercise Sciences, Department for Neuromotor Behavior and Exercise, Westfälische Wilhelms University Münster, Horstmarer Landweg 62b, 48149 Münster, Germany, nils.pixa@uni-muenster.de

^4^School of Psychology, Shanghai University of Sport, No. 650 Qingyuan Ring Road, Yangpu District, Shanghai, 200438, China, dylancheng@protonmail.com

#authors contributed equally to this work

***Corresponding author:** Fabian Steinberg, Louisiana State University, School of Kinesiology, email: fsteinberg@lsu.edu, Tel: +1 225-578-2923

**Table A.1.** Mean values (SDs) of all analyzed parameters of the flanker test for the baseline and online measures per experiment and condition

| Experiments | N | Condition | RT incon. Stimuli (ms) | | Accuracy (% corr.) | | Flanker effect (ms) | |
| --- | --- | --- | --- | --- | --- | --- | --- | --- |
|  |  |  | Baseline | Online | Baseline | Online | Baseline | Online |
| a-tDCS vs. s-tDCS d. AC (EXP. 1) | 21 | Control | 386.85 (41.05) | 375.85 (33.86) | 95.70 (3.00) | 95.65 (2.50) | 69.10 (18.80) | 67.75 (16.07) |
|  |  | Intervention | 390.85 (43.18) | 376.89 (36.47) | 95.79 (3.11) | 95.79 (3.15) | 72.29 (18.22) | 70.09 (22.92) |
| c-tDCS vs. s-tDCS d.AC (EXP. 2) | 17 | Control | 374.16 (36.08) | 359.41 (35.44) | 96.72 (3.26) | 96.10 (4.35) | 72.05 (14.31) | 62.78 (16.81) |
|  |  | Intervention | 364.17 (30.10) | 354.36 (28.36) | 95.25 (4.75) | 95.14 (4.00) | 63.06 (15.35) | 58.55 (17.43) |
| AE vs. AC (EXP. 3) | 23 | Control | 386.52 (58.22) | 374.78 (46.17) | 94.44 (5.75) | 94.77 (4.79) | 71.17 (29.40) | 73.08 (28.76) |
|  |  | Intervention | 383.13 (49.78) | 362.33 (45.64) | 95.03 (4.22) | 94.23 (4.56) | 73.81 (22.86) | 67.40 (18.64) |
| a-tDCS vs. s-tDCS d.AE (EXP. 4) | 16 | Control | 374.59 (39.24) | 359.47 (34.70) | 94.71 (3.77) | 94.29 (3.50) | 66.23 (13.38) | 62.17 (17.97) |
|  |  | Intervention | 374.11 (35.73) | 355.09 (32.82) | 95.55 (2.96) | 95.43 (2.73) | 68.37 (19.47) | 61.66 (18.28) |
| c-tDCS vs. s-tDCS d. AE (EXP. 5) | 24 | Control | 373.01 (29.15) | 344.65 (25.46) | 94.79 (3.45) | 94.55 (3.78) | 70.74 (22.52) | 63.25 (16.61) |
|  |  | Intervention | 369.04 (31.28) | 345.25 (31.86) | 95.31 (2.87) | 93.75 (4.10) | 68.27 (21.62) | 59.98 (19.90) |

**Table A.2.** p-values of the Shapiro-Wilk Test for normality

| Experiment | N | Condition | RT incon. Stimuli (ms) | | Accuracy (% corr.) | | Flanker effect (ms) | |
| --- | --- | --- | --- | --- | --- | --- | --- | --- |
|  |  |  | Baseline | Online | Baseline | Online | Baseline | Online |
| a-tDCS vs. s-tDCS d. AC (EXP. 1) | 21 | Control | .019 | .138 | .175 | .021 | .066 | .535 |
|  |  | Intervention | .11 | .522 | .35 | .189 | .579 | .345 |
| c-tDCS vs. s-tDCS d.AC (EXP. 2) | 17 | Control | .574 | .081 | .012 | < .001 | .227 | .126 |
|  |  | Intervention | .166 | .802 | .006 | < .001 | .088 | .169 |
| AE vs. AC (EXP. 3) | 23 | Control | .983 | .869 | < .001 | .017 | .026 | .467 |
|  |  | Intervention | .323 | .615 | .014 | .008 | .332 | .573 |
| a-tDCS vs. s-tDCS d.AE (EXP. 4) | 16 | Control | .219 | .075 | .165 | .522 | .98 | .344 |
|  |  | Intervention | .564 | .234 | .125 | .621 | .92 | .264 |
| c-tDCS vs. s-tDCS d. AE (EXP. 5) | 24 | Control | .137 | .605 | .077 | .052 | .622 | .148 |
|  |  | Intervention | .213 | .082 | .304 | .078 | .518 | .744 |

**Table A.3.** Main and interaction effects of the 2x2 rmANOVA for reaction times of incompatible stimuli for each EXP.

| Experiment | N |  | df | F | p | *η_p_^2^* |
| --- | --- | --- | --- | --- | --- | --- |
| a-tDCS vs. s-tDCS d. AC  (EXP. 1) | 21 | Time | 1, 20 | 14.3 | < .001 | .42 |
|  |  | Condition | 1, 20 | .18 | .68 | .01 |
|  |  | Time*Condition | 1, 20 | .5 | .49 | .02 |
| c-tDCS vs. s-tDCS d. AC  (EXP. 2) | 17 | Time | 1,16 | 32.32 | < .001 | .67 |
|  |  | Condition | 1,16 | 3.6 | .07 | .18 |
|  |  | Time*Condition | 1,16 | .83 | .36 | .05 |
| AE vs. AC  (EXP. 3) | 23 | Time | 1,22 | 28.45 | < .001 | .56 |
|  |  | Condition | 1,22 | 2 | .17 | .08 |
|  |  | Time*Condition | 1,22 | 2.75 | .112 | .11 |
| a- vs. s-tDCS d. AE  (EXP. 4) | 16 | Time | 1,15 | 27.9 | < .001 | .65 |
|  |  | Condition | 1,15 | .18 | .67 | .01 |
|  |  | Time*Condition | 1,15 | .56 | .47 | .04 |
| c- vs. s-tDCS d. AE  (EXP. 5) | 24 | Time | 1,23 | 118.67 | < .001 | .84 |
|  |  | Condition | 1,23 | .12 | .73 | .01 |
|  |  | Time*Condition | 1,23 | 1.84 | .189 | .07 |

**Table A.4.** Main and interaction effects of the 2x2 rmANOVA for response accuracy for each EXP.

| Experiment | N |  | df | F | p | *η_p_^2^* |
| --- | --- | --- | --- | --- | --- | --- |
| a-tDCS vs. s-tDCS d. AC  (EXP. 1) | 21 | Time | 1,20 | .0 | .96 | 0 |
|  |  | Condition | 1,20 | .07 | .79 | 0 |
|  |  | Time*Condition | 1,20 | .0 | .95 | 0 |
| c-tDCS vs. s-tDCS d. AC  (EXP. 2) | 17 | Time | 1,16 | .39 | .54 | .02 |
|  |  | Condition | 1,16 | 4.5 | .05 | .22 |
|  |  | Time*Condition | 1,16 | .15 | .7 | .01 |
| AE vs. AC  (EXP. 3) | 23 | Time | 1,22 | .45 | .5 | .02 |
|  |  | Condition | 1,22 | 0 | .97 | 0 |
|  |  | Time*Condition | 1,22 | .84 | .369 | .04 |
| a- vs. s-tDCS d. AE  (EXP. 4) | 16 | Time | 1,15 | .15 | .7 | .01 |
|  |  | Condition | 1,15 | 2.86 | .1 | .16 |
|  |  | Time*Condition | 1,15 | .15 | .7 | .01 |
| c- vs. s-tDCS d. AE  (EXP. 5) | 24 | Time | 1,23 | 2.87 | .1 | .1 |
|  |  | Condition | 1,23 | .05 | .83 | 0 |
|  |  | Time*Condition | 1,23 | 4.58 | .043 | .17 |

**Table A.5.** Main and interaction effects of the 2x2 rmANOVA for the flanker effect for each EXP.

| Experiment | N |  | df | F | p | *η_p_^2^* |
| --- | --- | --- | --- | --- | --- | --- |
| a-tDCS vs. s-tDCS d. AC  (EXP. 1) | 21 | Time | 1,20 | .47 | .05 | .02 |
|  |  | Condition | 1,20 | .63 | .44 | .03 |
|  |  | Time*Condition | 1,20 | .04 | .84 | .01 |
| c-tDCS vs. s-tDCS d. AC  (EXP. 2) | 17 | Time | 1,16 | 7.28 | .02 | .31 |
|  |  | Condition | 1,16 | 1.59 | .23 | .09 |
|  |  | Time*Condition | 1,16 | 1.10 | .32 | .06 |
| AE vs. AC  (EXP. 3) | 23 | Time | 1,22 | 1.00 | .33 | .04 |
|  |  | Condition | 1,22 | .15 | .70 | .01 |
|  |  | Time*Condition | 1,22 | 3.74 | .06 | .15 |
| a- vs. s-tDCS d. AE  (EXP. 4) | 16 | Time | 1,15 | 5.32 | .03 | .26 |
|  |  | Condition | 1,15 | .04 | .86 | .01 |
|  |  | Time*Condition | 1,15 | .22 | .65 | .01 |
| c- vs. s-tDCS d. AE  (EXP. 5) | 24 | Time | 1,23 | 16.96 | .01 | .42 |
|  |  | Condition | 1,23 | .68 | .42 | .03 |
|  |  | Time*Condition | 1,23 | .05 | .82 | .00 |

**Table A.6**. Main and interaction effects of the 2x2 rmANOVA for ratings of perceived exertion for each tDCS-AE EXP.

| Experiment | N |  | df | F | p | *η_p_^2^* |
| --- | --- | --- | --- | --- | --- | --- |
| a- vs. s-tDCS d. AE  (EXP. 4) | 16 | Time | 1.24, 21 | 4.38 | .04 | .21 |
|  |  | Condition | 1,15 | 1.54 | .02 | .08 |
|  |  | Time*Condition | 2.5, 42.44 | .082 | .47 | .05 |
| c- vs. s-tDCS d. AE  (EXP. 5) | 21 | Time | 1.63, 32.69 | .40 | .64 | .02 |
|  |  | Condition | 1,20 | .34 | .57 | .02 |
|  |  | Time*Condition | 2.61, 52.20 | .97 | .40 | .05 |

**Table A.7.** Main and interaction effects of the 2x2 rmANOVA for heart rate for each tDCS-AE EXP.

| Experiment | N |  | df | F | p | *η_p_^2^* |
| --- | --- | --- | --- | --- | --- | --- |
| a- vs. s-tDCS d. AE  (EXP. 4) | 16 | Time | 2.23, 37.85 | 2.87 | .07 | .14 |
|  |  | Condition | 1,15 | 1.08 | .31 | .06 |
|  |  | Time*Condition | 2.28, 38.76 | .56 | .60 | .03 |
| c- vs. s-tDCS d. AE  (EXP. 5) | 21 | Time | 1.30, 25.97 | 6.11 | .014 | .23 |
|  |  | Condition | 1,20 | 1.50 | .24 | .07 |
|  |  | Time*Condition | 1.97, 39.40 | .56 | .70 | .03 |

**Table A.8**. Main and interaction effects of the 2x2 rmANOVA for cycling resistance (Watt) for each tDCS-AE EXP

| Experiment | N |  | df | F | p | *η_p_^2^* |
| --- | --- | --- | --- | --- | --- | --- |
| a- vs. s-tDCS d. AE  (EXP. 4) | 16 | Time | 1.65, 28.04 | 40.20 | .00 | .70 |
|  |  | Condition | 1,15 | 1.04 | .03 | .06 |
|  |  | Time*Condition | 2.14, 36.39 | 2.78 | .07 | .14 |
| c- vs. s-tDCS d. AE  (EXP. 5) | 21 | Time | 1.25, 25.03 | 44.70 | .00 | .70 |
|  |  | Condition | 1,20 | .00 | .97 | .00 |
|  |  | Time*Condition | 1.76, 35.19 | .67 | .50 | .03 |
